# Effects of salivary protein flow and indigenous microorganisms on initial colonization of *Candida albicans* in an *in vivo* model

**DOI:** 10.1186/1472-6831-12-36

**Published:** 2012-08-31

**Authors:** Norihiko Kanaguchi, Naoki Narisawa, Tatsuro Ito, Yosuke Kinoshita, Yasuka Kusumoto, Osamu Shinozuka, Hidenobu Senpuku

**Affiliations:** 1 Dentistry for Persons with Disabilities, Tokyo Medical & Dental University, Tokyo, Japan; 2 Department of Pediatric Dentistry, Nihon University Graduate School of Dentistry at Matsudo, Chiba, Japan; 3 Department of Bacteriology, National Institute of Infectious Diseases, 1-23-1 Toyama, Shinjuku-ku, Tokyo, 162-8640, Japan

**Keywords:** Candida albicans, NOD/SCID.*e2f1*^-^ mice, Saliva, Colonization, Sucrose

## Abstract

**Background:**

*Candida albican*s is a dimorphic fungus that is part of the commensal microbial flora of the oral cavity. When the host immune defenses are impaired or when the normal microbial flora is disturbed, *C. albicans* triggers recurrent infections of the oral mucosa and tongue. Recently, we produced NOD/SCID.*e2f1*^-/-^ mice that show hyposalivation, decrease of salivary protein flow, lack IgA and IgG in saliva, and have decreased NK cells. Our objective was to characterize *C. albicans* infection and biofilm formation in mice*.*

**Methods:**

NOD/SCID.*e2f1*^-/-^ mice were used as an animal model for *C. albicans* infection. *C. albicans* yeast and hyphal forms solutions were introduced in the oral cavity after disinfection by Chlorhexidine.

**Results:**

The numbers of *C. albicans* colonized and decreased in a time-dependent manner in NOD/SCID*.e2f1*^+/+^ after inoculation. However, the colonization levels were higher in NOD/SCID*.e2f1*^+/+^ than NOD/SCID.*e2f1*^-/-^ mice. In the mice fed 1% sucrose water before inoculation, *C. albicans* sample was highly contaminated by indigenous microorganisms in the oral cavity; and was not in the mice fed no sucrose water. The colonization of *C. albicans* was not influenced by the contamination of indigenous microorganisms. The hyphal form of *C. albicans* restricted the restoration of indigenous microorganisms. The decreased saliva in NOD/SCID.*e2f1*^-/-^ did not increase the colonization of *C. albicans* in comparison to NOD/SCID*.e2f1*^+/+^ mice**.** We suggest that the receptor in saliva to *C. albicans* may not be sufficiently provided in the oral cavity of NOD/SCID.*e2f1*^-/-^ mice.

**Conclusion:**

The saliva protein flow may be very important for *C. albicans* initial colonization, where the indigenous microorganisms do not affect colonization in the oral cavity*.*

## Background

*Candida* spp*.* are human commensals commonly colonizing human mucosal surfaces and can participate in biofilm formation on mucosal and cutaneous surfaces as well as on device surfaces, e.g., dentures, venous catheters, and urinary catheters [1-7]. However, under certain conditions, the fungus causes a variety of infections from mild mucosal infections to life-threatening invasive candidiasis [8]. Many manifestations of candidiasis are associated with the formation of biofilms on host tissues (e.g., oral thrush) and on indwelling medical devices (central venous catheter-associated candidemia) [9]. A clinically significant characteristic of microbial biofilms is their enhanced resistance to antimicrobial drug therapies [10-12].

*Candida albican*s is commensal on human mucosal surfaces and is one of the most important nosocomial infections of humans. *C. albicans* is the most common cause of various forms of candidiasis [13]. Under conditions of immune dysfunction, colonizing *C. albicans* can become an opportunistic pathogen causing recurrent mucosal infections of the oral cavity and vagina in immune-compromised hosts such as the elderly and HIV-positive patients [14,15]. *C. albicans* is a pleiomorphic fungus having the ability to transition between cellular yeast and filamentous forms essential for virulence; therefore filamentous forms constitute a central component in *C. albicans* pathogenesis. Various models have been established to study candidiasis, including *Candida*-epithelial cell interactions using immortalized human epithelial cell lines in tissue culture [16-18] where *C. albicans* adheres to the epithelial cells of the oral mucosa and also invades these cells. Invasion into the epithelial cell limit of the oral mucosa is characteristic of both human and experimental animal models of oropharygeal candidiasis [19-22].

A recent investigation developed NOD/SCID background E2F-1 deficient mice (NOD/SCID.*e2f1*^−/−^) (T and B cells do not develop where the E2F-1 function loss in the NOD/SCID background mice do not have an auto-reactive response) [23]. The NOD/SCID.*e2f1*^−/−^ mice have decreased saliva volume, lack sIgA and IgG in the saliva, as compared with NOD.*e2f1*^+/+^ mice. This mouse have decreased NK cells as compared with SCID mice and may be a useful animal model for studying oral infection, colonization, and biofilm formation. Further, human tissue grafts can be used in this mouse. These mice have long survival because they do not develop systemic diseases such as Insulin Dependent Diabetes Mellitus and Sjögren's Syndrome in comparison to the parent strain NOD*.e2f1*^−/−^ mice. Recently, we found these mice are highly susceptible to dental caries pathogen; *Streptococcus mutans* colonization when NOD/SCID.*e2f1*^−/−^ mice are pre-treated with human saliva or sIgA using a low concentration (1%) sucrose supplement; *S. mutans* biofilm formation occurred when the mice were supplied 1% sucrose water and a non-sucrose diet [24]. Therefore, these mice may be a useful animal model for *C. albicans* infections in the oral cavity because they have decreased saliva volume and are impaired in immune activity by T, B cells and NK cells.

Mammalian infection models, in particular mouse models, are commonly used to study host-pathogen interaction of human pathogens. Murine models for both superficial and systemic *C. albicans* infections have been developed and are widely used to study pathogenesis and to determine the virulence of defined *C. albicans* mutants [25,26]. Saliva includes various anti-microbial agents and is likely to play important roles in resistance to infection by *C. albicans* in the oral cavity. Immune-deficient mice such as Rag1^−/−^ and CB-17.SCID mice have been used as an infection model for *C. albicans *[27]. However, very little is known about the contribution of saliva in initiation of *C. albicans* colonization and mucosal infection in the oral cavity using the mouse model. In this study, we investigated the initial colonization of *C. albicans* in the animal model using NOD/SCID.*e2f1*^+/+^ and NOD/SCID.*e2f1*^−/−^ mice.

## Methods

### Strains and culture conditions

**Figure 1:**
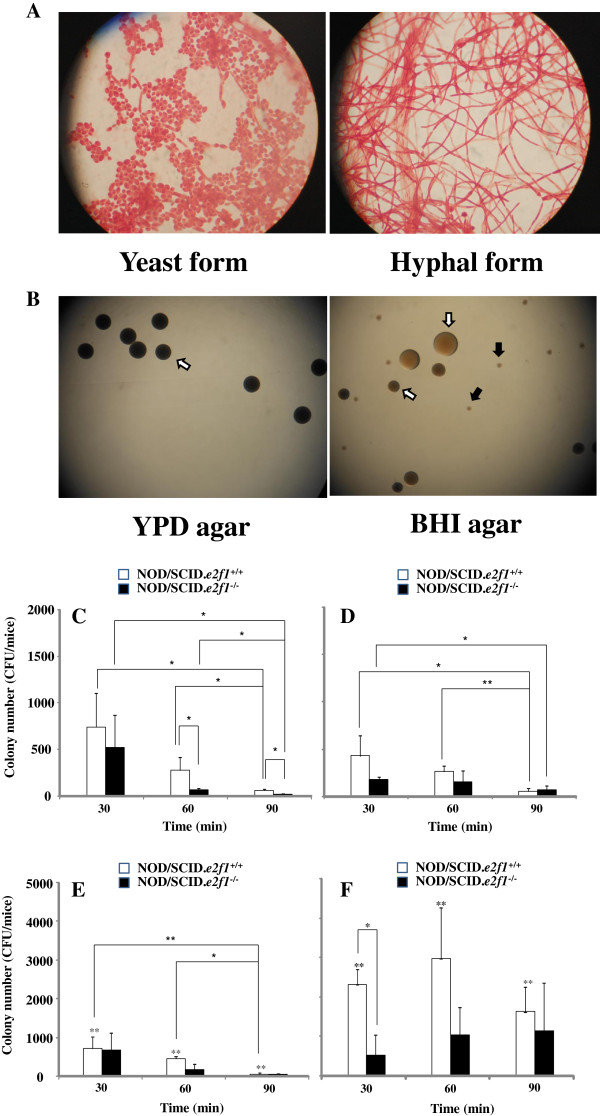
**Observation of *C. albicans *yeast and hyphal forms, and colony numbers after inoculation. ***C. albicans* yeast and hyphal forms were taken pictures in microscope after incubation of them in YPD and RPMI1640 with 5% FBS (**A**). The colonization was observed on the YPD agar and BHI agar plates poured swab samples from oral cavity of NOD/SCID.*e2f1*^−/−^ mice fed 1% sucrose water at 60 min after the inoculation (**B**). White and black arrow indicated *C. albicans* and indigenous microorganism colony respectively. These colonies were taken pictures in the stereoscopic microscope. Data were representative in three independent assays. Samples were swabbed from the oral cavities of four 4-month-old female NOD/SCID.*e2f1*^+/+^ and NOD/SCID.*e2f1*^−/−^ mice at 30, 60, and 90 minutes after inoculation using the yeast form. Samples swabbed in mice fed water (**C**) or 1% sucrose water (**D**) overnight before inoculation were poured on YPD agar. Samples swabbed in mice fed water (**E**) or 1% water (**F**) before inoculation were poured on BHI agar. CFU data were obtained from three independent experiments with four mice from each strain. Values are expressed as the means ± standard deviations (SDs) of the data. Asterisks show significant differences (*P <0.05) in C and D and significant differences (** <0.05) between water (**E**) and 1% sucrose water (**F**) for drinking.

The strain used was *C. albicans* SC5314. *C. albicans* was incubated for 24 h at 37°C in Yeast Peptone Dextrose (YPD; 2% Bacto peptone, 2% dextrose and 1% yeast extract, Becton Deckinson, Sparks, MD) broth before the beginning of each experiment. The yeast form of *C. albicans* was predominant in culture with YPD overnight; whereas the mycelial form occurred in RPMI1640 (Gibco-Invitrogen, Grand Island, NY) with 5% FBS in overnight culture (Figure 1A). The two cell types were used to inoculate the oral cavity.

### Animals

Heterozygous ΔE2F-1 NOD/SCID mice were bred to generate homozygous ΔE2F1 NOD/SCID mice. Three strains of NOD/SCID mice (*e2f1* +/+, +/− and −/−) were identified using PCR [23]. All mice used were female, 4 months of age, and were maintained in accordance with the guidelines for the Care and Use of Laboratory Animals from the National Institute of Infectious Diseases. Experimental protocols (#211125) were approved by the National Institute of Infectious Diseases Animal Resource Committee.

### Bacterial sampling and colony-forming unit (CFU) estimates

Bacterial inoculation, sampling, and CFU estimates were performed using procedures and conditions described previously [28-30]. *C. albicans* yeast and hyphal forms were cultured in YPD and RPMI1640 with 5% FBS broth overnight and washed twice with sterile phosphate-buffered saline (PBS). In our previous study we demonstrated *S. mutans* colony counts were significantly higher than that of other streptococci (i.e. *S. sanguis, S. sobrinus,* and *S. salivarius*) in mice that ingested 1% sucrose in water one day before inoculation [28]. In the same way, NOD/SCID.*e2f1*^+/+^ and NOD/SCID.*e2f1*^−/−^ mice were also given drinking water containing 1% sucrose (less than the usual concentration in juice) one day prior to *C. albicans* inoculation to encourage the early adherence of *C. albicans* in conditions resembling a natural oral environment. In contrast, drinking water (no sucrose) was used as a control. Chlorhexidine (CHX; 0.2%) soaked sterile cotton swabs were used to disinfect the oral cavities of the mice including teeth, tongue, and mucosal surfaces. The cavity was immediately washed with sterile PBS. This disinfection was confirmed as few bacterial cells were observed in mice at 30, 60, and 90 min after the inoculation. *C. albicans* yeast and hyphal forms solutions were introduced to the oral cavities of three females at 4 months of age at a final concentration of 5 × 10^9^CFU in 250 μl of PBS during a 1 min period. Following inoculation, samples were collected from the tongue and buccal mucosa with a sterile cotton ball at 30, 60 and 90 min after the inoculation and then dipped in 2 ml of PBS. The samples in sterile PBS were sonicated using ultrasonic dispersion (power output, 60 W) for 10 s, and then poured onto YPD agar to determine *C. albicans* colony number and on BHI agar for *C. albicans* and indigenous microorganism colony number (Figure 1B). CFUs were determined by counting colonies on the YPD agar after 24 hours and on BHI agar plates after 48 h using aerobic incubation at 37°C.

### Statistical analysis

All data were analyzed using the Statistical Package for SPSS for Windows (version 100, Chicago, IL). The Student’s t-test was used to compare control data and the data for *C. albicans* after incubation. * indicate *P*-values less than 0.05, and ** at 0.01 were considered to be significant.

## Results

### Effects of decreasing saliva and indigenous oral microorganisms on the *C. albicans* initial colonization

Saliva is thought to play a significant role in the attachment and colonization of *C. albicans* on tooth and oral mucosa. We evaluated the effects of different volumes of saliva on *C. albicans* colonization using NOD/SCID wild type and NOD/SCID.*e2f1*^−/−^ mice. The CFU numbers of *C. albicans* yeast form colonized in the oral cavity after disinfection by chlorhexidine. *C. albicans* colonization in both mice were significantly decreased from 30 min to 90 min after inoculation in NOD/SCID.*e2f1*^+/+^ mice when they were treated with or without sucrose water (Figure 1C and D). The colonization was also decreased; but these reductions were not significantly different in NOD/SCID.*e2f1*^−/−^ mice as compared to NOD/SCID.*e2f1*^+/+^ mice. *C. albicans* CFU numbers were significantly lower in NOD/SCID.*e2f1*^−/−^ mice than those in NOD/SCID wild type mice fed water after 60 and 90 min post inoculation (Figure 1C). Therefore, decreasing saliva is associated with reduced colonization of *C. albicans*. However, the significant differences between NOD/SCID wild and NOD/SCID.*e2f1*^−/−^ mice disappeared by including sucrose in the drinking water (Figure 1D). YPD was used as a selective medium for *C. albicans* because there was a possibility that some indigenous microorganisms in mice may contaminate the swabbed sample after disinfection by Chlorhexidine*.* BHI agar plating was also used to confirm contamination levels by indigenous microorganisms. The total number of *C. albicans* and indigenous microorganisms on the BHI agar were similar to those on the YPD plates in the NOD/SCID.*e2f1*^+/+^ mice with no-sucrose drinking water and the time-dependent decreasing was also similar in colony numbers in YPD to those in BHI (Figure 1C and Figure 1E). However, the total number of *C. albicans* and indigenous microorganisms increased significantly in the sucrose-water as compared with no-sucrose water in NODSCID.*e2f1*^+/+^ mice (Figure 1E and F). However, there were no significant differences among samples at each sampling time in the drinking of sucrose water. In the case of NOD/SCID.*e2f1*^−/−^ mice, a few indigenous bacteria were increased significantly by adding of sucrose in the drinking water, and the colonization did not show significant differences among sampling times (Figure 1E and F). In contrast, with sucrose in the drinking water, many indigenous microorganisms were found because CFU numbers on BHI were significantly higher than those on YPD using the same sample (Figure 1F). Therefore, sucrose helps time-dependent restoration of indigenous microorganisms after disinfection by chlorhexidine but does not affect increased infection by *C. albicans*. Further, decreasing saliva may not help the colonization of *C. albicans* or restoration of indigenous bacteria in NOD/SCID.*e2f1*^−/−^ mouse system*.*

### Comparison of colonization between yeast and hyphal forms in mouse oral cavity

**Figure 2:**
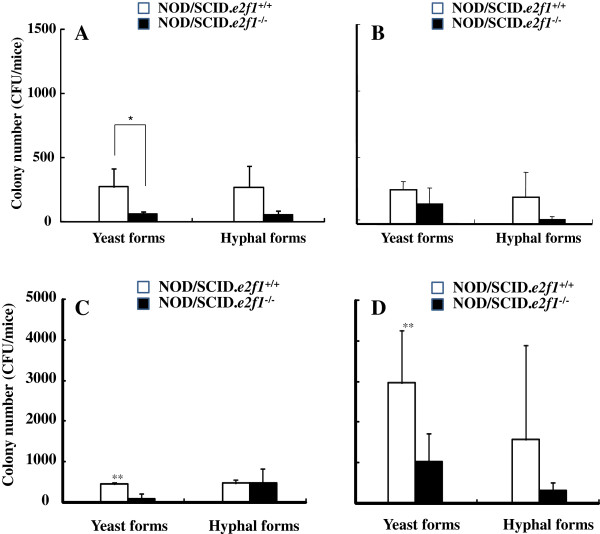
**Comparison of the yeast form and hyphal form in colony numbers of *C. albicans. ***Samples swabbed from the oral cavities at 60 minutes after inoculation of the yeast form or hyphal form in four 4-month-old female NOD/SCID.*e2f1*^+/+^ and NOD/SCID.*e2f1*^−/−^ mice fed water (**A**) or 1% sucrose water (**B**) overnight were poured on YPD agar. Other samples in mice fed water (**C**) or 1% sucrose water (**D**) were poured on BHI agar. CFU data were obtained from three independent experiments with four mice from each strain, and values are expressed as the means ± standard deviations (SDs) of the data. Asterisks show significant differences (*P <0.05) in A and significant differences (** <0.05) between water (**C**) and 1% sucrose water (**D**) for drinking.

In severe infection by *C. albicans*, the hyphal form has very important roles as compared to the yeast form. Therefore we tested oral colonization of the hyphal form compared to the yeast form at 60 min after inoculation. The colonizations of *C. albicans* were similar in the hyphal and yeast forms in both mice (Figure 2A and B). The colonization of total microorganisms was significantly higher with sucrose drinking water than with no-sucrose water in the yeast form, but there was no difference in the hyphal form in NOD/SCID*.e2f1*^+/+^ mice (Figure 2C and D). The decreased saliva volume in NOD/SCID.*e2f1*^−/−^ mice and hyphal form in both mice did not induce the effects of sucrose drinking water on the ratio of the yeast form in NOD/SCID.*e2f1*^+/+^ mice. Therefore, the saliva volume and hyphal form of *C. albicans* may restrict the restoration of indigenous microorganisms.

## Discussion

The colonization of *C. albicans* depends on several factors: the acquisition or entry of cells into the oral cavity, the attachment and growth of these cells, the penetration of tissues, and the removal of cells from the oral cavity. In this study, the NOD/SCID.*e2f1*^−/−^ mice with decrease of saliva showed negative colonization of *C. albicans* as compared with NOD/SCID wild type mice. On the tooth surface coated with a salivary pellicle microbial adherence interactions may involve adsorbed saliva molecules. The saliva pellicle increases the adherence of *C. albicans* cells to HA beads [31]. Other investigators previously have shown the presence of serum and salivary pellicles can help *C. albicans* colonization on acylic strips and denture lining materials [32,33]. Therefore, we speculate the NOD/SCID mice produce saliva containing receptors to bind to *C. albicans* and create a saliva-exposing environmental mucosal surface in the oral cavity.

In contrast, innate defenses include the epithelial barrier and anti-candidal compounds in saliva such as lysozyme [34], histatins [35], lactoferrin [36], and calprotectin [37,38]. Innate host defenses are critical in the maintenance of oral health. Saliva includes lysozome, lactoferrin, and histatins that are thought to be the three major non-immunological antimicrobial proteins to modulate *Candida* populations in the oral cavity [39]. However, the decreased saliva negatively modulated *C. albicans* populations in this study. In our previous study, the saliva from NOD/SCID.*e2f1*^+/+^ and *e2f1*^−/−^ mice was characterized in protein concentration/ml, amylase activities, and protein concentration per min/ml [24]. There were no significant differences in protein concentration and amylase activity between the mice. The protein concentration per min in 1 ml saliva was significantly lower in NOD/SCID.*e2f1*^−/−^ mice as compared to NOD/SCID.*e2f1*^+/+^ mice. Here we did not measure the activities of lysozyme, lactoferrin, or histatins in saliva from NOD/SCID*.e2f1*^+/+^ mice and NOD/SCID*.e2f1*^−/−^ mice, but the activities of the innate immunity and the receptors to *C. albicans* attachment may not be sufficiently provided in oral cavity from NOD/SCID.*e2f1*^−/−^ as compared with NOD/SCID*.e2f1*^+/+^ mice. Mouse salivary proteins are poorly existed in NOD/SCID.*e2f1*^−/−^ mice. Taken together, mouse saliva works positively for the initial colonization of *C. albicans* rather than protecting by innate immunity, an opposite function to *S. mutans* colonization [24].

The colonization decreased in a time-dependent manner without the effects of saliva volume difference in NOD/SCID.*e2f1*^+/+^ and NOD/SCID.*e2f1*^−/−^ mice. The colonization may be decreased by washing effects with an appropriate volume of saliva. Moreover, cell-mediated immunity plays an important role in the resistance to mucosal candidiasis [40,41]. The mucosal surface provides a protective barrier against bacterial and fungal infections in the oral cavity. The β-defensins are small cationic amphipathic peptides that exhibit a broad spectrum of activity against gram-positive and gram-negative bacteria and fungal species [42]. NOD/SCID.*e2f1*^+/+^ and NOD/SCID.*e2f1*^−/−^ mice do not have mature T and B cells but have a protective barrier with cell-mediated immunity. Therefore, cell-mediated immunity may not be associate with the difference of *C. albicans* colonization between NOD/SCID.*e2f1*^+/+^ and NOD/SCID.*e2f1*^−/−^ mice. The positive function for *C. albicans* in the oral cavity indicates a useful animal model for initial colonization of *C. albicans* in NOD/SCID wild type mice as compared to NOD/SCID*.e2f1*^−/−^ mice.

Previous report suggested that the indigenous bacterial flora could suppress the extent of colonization of *C. albicans* by interfering with its ability to attach to mucosal surface in comparison with germ-free rats and conventional rat [43]. This was explained by competing for epithelial receptor sites required for Candida attachment or by enzymatically altering the surfaces of the yeast cells. Our mouse model system using oral inoculation of *C. albicans* is different from previous report using germ free and conventional rat and indicates better model to explore effects of salivary components and indigenous microorganisms on the colonization under the natural background condition than previous model system. Sucrose works as a substrate for production of glucan by oral streptococci. The drinking of sucrose water before inoculation provides a source of glucan for the restoration of indigenous microorganisms. CHX was uniformly effective against strains of common borne microorganisms. The treatment with CHX disinfected the indigenous microorganism before inoculation in both no-sucrose and sucrose drinking water [44,45]. However, the indigenous microorganisms contaminated the sample of *C. albicans* in the mice fed 1% sucrose water. The colonization of *C. albicans* was not affected by restoration of indigenous microorganisms. Therefore, the initial colonization of *C. albicans* is not affected by later colonization of indigenous microorganisms in the oral cavity. It is suggested that the colonized cells of *C. albicans* are not removed by the enzymatical or physical effect by indigenous microorganisms.

Yeast-form cells adhere more effectively than hypal cells to endothelial cells under conditions of flow [46]. Although cells locked in the filamentous state also display reduced virulence [47-49], the ability to form hyphae is important for *C. albicans* to cause disease after dissemination: cells locked in the yeast form remain avirulent until they are permitted to form hyphae, after which, mice succumb to the infection [50]. *C. albicans* hyphae are impaired in their ability to adhere to the human oral cavity by the bacterium *Streptococcus gordonii *[51]. To colonize and infect the oral environment, yeast cells must first adhere to host cells and tissues or prosthetic materials within the oral cavity or co-aggregate with the oral microbiota [52-54]. In this study, the hyphae form of *C. albicans* showed similar results as the yeast form but the restoration of the indigenous microorganisms was weaker with the hyphal form than that with the yeast form. This may indicate restriction effects by the hyphal form on the restoration.

We provide here an original new animal model system, which may be a useful model to explore oral initial colonization of *C. albicans.* The salivary protein flow may play important roles in maintaining the commensal behavior of *C. albicans* and becoming an opportunistic pathogen under the immune deficiency condition such as SCID. These mice are required for these investigations to determine several factors that contribute to the susceptibility for candidal infections.

## Conclusions

In conclusion, the saliva protein flow may be very important for *C. albicans* initial colonization, where the indigenous microorganisms do not affect *C. albicans* initial colonization in the oral cavity*.*

## Competing interests

The authors declare that they have no competing interests.

## Authors’ contributions

NK carried out animal experiments and attend to draft the manuscript. NN helped animal experiments and to draft the manuscript. TI helped to produce mouse. YK helped to maintain and produce mouse. YK participated in the design of the study. OS participated in the design of study and coordination. HS designed the study, carried out production of mouse and described manuscript. All authors read and approved the final manuscript.

## Pre-publication history

The pre-publication history for this paper can be accessed here:

http://www.biomedcentral.com/1472-6831/12/36/prepub

## References

[B1] Douglass LJ *Candida* biofilms and their role in infection Trends Microbiol 2003 11 30 36 10.1016/S0966-842X(02)00002-1 12526852

[B2] Douglass LJ Medical importance of biofilms in *Candida* infections Rev Iberoam Micol 2002 19 139 143 12825991

[B3] Kumamoto CA *Candida* biofilms Curr Opin Microbiol 2002 5 608 611 10.1016/S1369-5274(02)00371-5 12457706

[B4] Kawamura-Sato K Wachino J Kondo T Ito H Arakawa Y Reduction of disinfectant bactericidal activities in clinically isolated *Actinetobacter* species in the presence of organic material J Antimicrob Chemother 2008 61 568 576 10.1093/jac/dkm498 18192683

[B5] Lopez-Ribot JL McAtee RK Perea S Kirkpatrick WP Rinalci MG Petterson TF Multiple resistance phenotypes of *Candida albicans* coexist during episodes of oropharyngeal candidiasis in human immunodeficiency virus-infected patients Antimicrob Agents Chemother 1999 43 1621 1630 10390213 10.1128/aac.43.7.1621 PMC89334

[B6] Mukherjee PK Clandra J *Candida* biofilm resistance Drug Resist Updat 2004 7 301 309 10.1016/j.drup.2004.09.002 15533767

[B7] Ramage G Wickes BL Lopez-Ribot JL Biofilms of *Candida albicans* and their associated resistance to antifungal agents Am Clin Lab 2001 20 42 44 11570274

[B8] Eggimann P Garbino J Pittet D Epidemiology of Candida species infections in critically ill non-immunosuppressed patients Lancet Infect Dis 2003 3 685 702 10.1016/S1473-3099(03)00801-6 14592598

[B9] Bailli GS Douglas LJ Matrix polymers of Candida biofilmsand their possible role in biofilm resistance to antifungal agents J Antimicrob Chemother 2000 46 397 403 10.1093/jac/46.3.397 10980166

[B10] Donlan RM Costerton JW Bioiofilms: survival mechanisms of clinically relevant microorganisms Clin Microbiol Rev 2002 15 167 193 10.1128/CMR.15.2.167-193.2002 11932229 PMC118068

[B11] Kumamoto CA Vinces MD Alternative *Candida albicans* lifestyles: growth on surfaces Annu Rev Microbiol 2005 59 113 133 10.1146/annurev.micro.59.030804.121034 16153165

[B12] Nett J Lincoln L Marchillo K Massey R Holoyda K Hoff B VanHandel M Andes D Putative role of beta-1,3 glucans in *Candida albicans* biofilm resistance Antimicrob Agents Chemother 2007 51 510 520 10.1128/AAC.01056-06 17130296 PMC1797745

[B13] Richards MJ Edwards JR Culver DH Gaynes RP Nosocomial infections in coronary care units in the United States. National Nosocomial Infections Surveillance System Am J Cardiol 1998 82 789 793 10.1016/S0002-9149(98)00450-0 9761092

[B14] Reichart PA Philipsen HP Schmidt-Westhausen A Samaranayake LP Pseudomembranous oral candidiasis in HIV infection: ultrastructural findings J Oral Pathol Med 1995 24 268 281 10.1111/j.1600-0714.1995.tb01182.x 7562665

[B15] de Repentigny L Lewandowski D Jolicoeur P Immunopathogenesis of oropharyngeal candidiasis in human immunodeficiency virus infection Clin Microbiol Rev 2004 17 729 759 10.1128/CMR.17.4.729-759.2004 15489345 PMC523562

[B16] Fu Y Rieg G Fonzi WA Belanger PH Edwards JE Filler SG Expression of the *Candida albicans* gene ALSI in *Saccharomyces cerevisiae* induces adherence to endothelial cells Infect Immun 1998 66 1783 1786 9529114 10.1128/iai.66.4.1783-1786.1998 PMC108121

[B17] McDonnell GE Antisepsis, Disinfection, and sterilization; types, action, and resistance 2007 Washington, DC: AZM press

[B18] Sandovsky-Losica H Chauhan N Calderone R Segal E Gene transcription studies of *Candida albicans* following infection of HEp2 epithelial cells Med Mycol 2006 44 329 334 10.1080/13693780500434701 16772226

[B19] Cawson RA Rajasingham KC Ultrastructural features of the invasive phase of *Candida albicans* Br J Dermatol 1972 87 435 443 10.1111/j.1365-2133.1972.tb01591.x 4567096

[B20] Eversole LR Reichart PA Ficarra G Schimidt-Westhausen A Romagnoli P Pimpinelli N Oral keratinocyte immune responses in HIV-associated candidiasis Oral Surg Oral Med Oral Pathol Oral Radiol Endodont 1997 84 372 380 10.1016/S1079-2104(97)90035-4 9347501

[B21] Kamai Y Kobota M Hosokawa T Fukuoka T Filler SG New model of oropharyngeal candidiasis in mice Antimicrob Agents Chemother 2001 45 3195 3197 10.1128/AAC.45.11.3195-3197.2001 11600377 PMC90803

[B22] Montes LF Wilbom WH Ultrastructural features of host-parasite relationship in oral candidiasis J Bacteriol 1986 96 1349 1356 5686004 10.1128/jb.96.4.1349-1356.1968 PMC252453

[B23] Matsui-Inohara H Uematsu H Narita T Satoh K Yonezawa H Kuroda K Ito T Yoneda S Kawarai T Sugiya H Watanabe H Senpuku H E2F-1-deficient NOD/SCID mice developed showing decreased saliva production Exp Biol Med 2009 234 1519 1524 10.3181/0903-RM-115 19934373

[B24] Ito T Maeda T Senpuku H Roles of salivary components in *Streptococcus mutans* colonization in a new animal model using NOD/SCID.e2f1−/− mice PLoS Onein press 10.1371/journal.pone.0032063 PMC3283720 22363797

[B25] Naglik JR Fidel PL Jr Odds FC Animal models of mucosal Candida infection FEMS Microbiol Lett 2008 283 129 139 10.1111/j.1574-6968.2008.01160.x 18422625 PMC3244615

[B26] de Repentigny L Animal models in the analysis of Candida host-pathogen interactions Curr Opin Microbiol 2004 7 324 329 10.1016/j.mib.2004.06.001 15288620

[B27] Pandiyan P Conti HR Zheng L Peterson AC Mathern DR Hernández-Santos N Edgerton M Gaffen SL Lenardo MJ Cell: CD4(+)CD25(+)Foxp3(+) regulatory T cells promote Th17 cells in vitro and enhance host resistance in mouse Candida albicans Th17 cell infection model Immunity 2011 34 422 434 10.1016/j.immuni.2011.03.002 21435589 PMC3258585

[B28] Matsumoto N Salam MA Watanabe H Amagasa T Senpuku H Role of gene *E2f1* in susceptibility to bacterial adherence of oral streptococci to tooth surfaces in mice Oral Microbiol Immunol 2004 19 270 276 10.1111/j.1399-302X.2004.00151.x 15209999

[B29] Senpuku H Matin K Salam MA Kurauchi I Sakurai S Kawashima M Murata T Miyazaki H Hanada N Inhibitory effects of MoAbs against a surface protein antigen in real-time adherence in vitro and recolonization in vivo of *Streptococcus mutans* Scand J Immunol 2001 54 109 116 10.1046/j.1365-3083.2001.00962.x 11439156

[B30] Salam MA Matsumoto N Matin K Tsuha Y Nakao R Hanada N Senpuku H Clin Diagn Lab Immunol 2004 11 379 386 15013991 10.1128/CDLI.11.2.379-386.2004 PMC371204

[B31] Cannon RD Nand AK Jenkinson HF Adherence of *Candida albicans* to human salivary components adsorbed to hydroxylapatite Microbiology 1995 141 213 219 10.1099/00221287-141-1-213 7894715

[B32] Nikawa H Nishimura H Hamada T Yamashiro H Samaranayake LP Effects of modified pellicles on Candida biofilm formation on acrylic surfaces Mycoses 1999 42 37 40 10.1046/j.1439-0507.1999.00270.x 10394846

[B33] Nikawa H Nishimura H Makihira S Hamada T Sadamori S Samaranayake LP Effect of serum concentration on Candida biofilm formation on acrylic surfaces Mycoses 2000 43 139 143 10.1046/j.1439-0507.2000.00564.x 10907344

[B34] Tobgi RS Samaranayake LP MacFarlane TW In vitro susceptibility of Candida species to lysozyme Oral Microbiol Immunol 1988 3 35 39 10.1111/j.1399-302X.1988.tb00603.x 3268748

[B35] Xu T Levitz SM Diamond RD Oppenheim FG Anticandidal activity of major human salivary histatins Infect Immun 1991 59 2549 2554 1855975 10.1128/iai.59.8.2549-2554.1991 PMC258054

[B36] Nikawa H Samaranayake LP Tenovuo J Pang KM Hamada T The fungicidal effect of human lactoferrin on *Candida albicans* and *Candida krusei* Arch Oral Biol 1993 38 1057 1063 10.1016/0003-9969(93)90167-K 8141667

[B37] Müller F Frøland SS Brandtzaeg P Fagerhol MK Oral candidiasis is associated with low levels of parotid calprotectin in individuals with infection due to human immunodeficiency virus Clin Infect Dis 1993 16 301 302 10.1093/clind/16.2.301 8443314

[B38] Challacombe SJ Immunologic aspects of oral candidiasis Oral Surg Oral Med Oral Pathol 1994 78 202 210 10.1016/0030-4220(94)90148-1 7936590

[B39] Samaranayake YH MacFarlane TW Samaranayake LP Aitchison TC The in vitro lysozyme susceptibility of Candida species cultured in sucrose supplemented media J Nat Prod 1992 55 1648 1654 10.1021/np50089a014 8336552

[B40] Cantorna MT Balish E Mucosal and systemic candidiasis in congenitally immunodeficient mice Infect Immun 1990 58 1093 1100 2180820 10.1128/iai.58.4.1093-1100.1990 PMC258587

[B41] Wei XQ Charles IG Smith A Ure J Feng GJ Huang FP Xu D Muller W Moncada S Liew FY Altered immune responses in mice lacking inducible nitric oxide synthase Nature 1995 375 408 411 10.1038/375408a0 7539113

[B42] Schutte BC Mitros JP Bartlett JA Walters JD Jia HP Welsh MJ Casavant TL McCray PB Jr Discovery of five conserved beta-defensin gene clusters using a computational search strategy Proc Natl Acad Sci U S A 2002 99 2129 2133 10.1073/pnas.042692699 11854508 PMC122330

[B43] Liljemark WF Gibbons RJ Suppression of *Candida albicans* by human oral streptococci in gnotobiotic mice Infect Immun 1973 8 846 849 4584056 10.1128/iai.8.5.846-849.1973 PMC422937

[B44] Müller G Kramer A Biocompatibility index of antiseptic agents by parallel assessment of antimicrobial activity and cellular cytotoxicity J Antimicrobial Chemother 2008 61 1281 1287 10.1093/jac/dkn125 18364400

[B45] Sanchez IR Nusbaum KE Swaim SF Hale AS Henderson RA McGuire JA Chlorhexidine diacetate and povidone-iodine cytotoxicity to canine embryonic fibroblasts Staphylococcus aureus 1988 17 182 185 10.1111/j.1532-950x.1988.tb00995.x 3238890

[B46] Grubb SE Murdoch C Sudbery PE Saville SP Lopez-Ribot JL Thornhill MH Adhesion of *Candida albicans* to endothelial cells under physiological conditions of flow Infect Immun 2009 77 3872 3878 10.1128/IAI.00518-09 19581400 PMC2738003

[B47] Braun BR Johnson AD Control of filament formation in *Candida albicans* by the transcriptional repressor TUP1 Science 1997 277 105 109 10.1126/science.277.5322.105 9204892

[B48] Braun BR Head WS Wang MX Johnson AD Identification and characterization of TUP1-regulated genes in *Candida albicans* Genetics 2000 156 31 44 10978273 10.1093/genetics/156.1.31 PMC1461230

[B49] Murad AM Leng P Straffon M Wishart J Macaskill S MacCallum D Schnell N Talibi D Marechal D Tekaia F d’Enfert C Gaillardin C Odds FC Brown AJ NRG1 represses yeast-hypha morphogenesis and hypha-specific gene expression in *Candida albicans* EMBO J 2001 20 4742 4752 10.1093/emboj/20.17.4742 11532938 PMC125592

[B50] Saville SP Lazzell AL Monteagudo C Lopez-Ribot JL Engineered control of cell morphology in vivo reveals distinct roles for yeast and filamentous forms of *Candida albicans* during infection Eukaryot Cell 2003 2 1053 1060 10.1128/EC.2.5.1053-1060.2003 14555488 PMC219382

[B51] Silverman RJ Nobbs AH Vickerman MM Barbour ME Jenkinson HF Interaction of Candida albicans cell wall Als3 protein with *Streptococcus gordonii* SspB adhesin promotes development of mixed-species communities Infect Immun 2010 78 4644 4652 10.1128/IAI.00685-10 20805332 PMC2976310

[B52] Cannon RD Chaffin WL Oral colonization by *Candida albicans* Crit Rev Oral Biol Med 1999 10 359 383 10.1177/10454411990100030701 10759414

[B53] Jabra-Rizk MA Falkler WA Jr Merz WG Kelley JI Baqui AA Meiller TF Coaggregation of *Candida dubliniensis* with *Fusobacterium nucleatum* J Clin Microbiol 1999 37 1464 1468 10203506 10.1128/jcm.37.5.1464-1468.1999 PMC84803

[B54] Chaffin WL López-Ribot JL Casanova M Gozalbo D Martínez JP Cell wall and secreted proteins of *Candida albicans*: identification, function, and expression Microbiol Mol Biol Rev 1998 62 130 180 9529890 10.1128/mmbr.62.1.130-180.1998 PMC98909

